# Blood pressure percentiles by age and body mass index for adults

**DOI:** 10.17179/excli2014-635

**Published:** 2015-03-24

**Authors:** Mostafa Hosseini, Masoud Baikpour, Mahmoud Yousefifard, Mohammad Fayaz, Jalil Koohpayehzadeh, Parisa Ghelichkhani, Hadi Asady, Fereshteh Asgari, Koorosh Etemad, Ali Rafei, Mohammad Mehdi Gouya

**Affiliations:** 1 Department of Epidemiology and Biostatistics, School of Public Health, Tehran University of Medical Sciences, Tehran, Iran; 2 Department of Medicine, School of Medicine, Tehran University of Medical Sciences, Tehran, Iran; 3 Department of Physiology, School of Medicine, Tehran University of Medical Sciences, Tehran, Iran; 4 Department of Community Medicine, Iran University of Medical Sciences, Tehran, Iran; 5 Saveh Medical University, Saveh, Iran; 6 Center for Disease Control, Ministry of Health and Medical Education, Tehran, Iran; 7 Department of Intensive Care Nursing, School of Nursing and Midwifery, Tehran, University of Medical Sciences, Tehran, Iran; 8 Department of Occupational Health Engineering, Faculty of Public Health, Tehran, University of Medical Sciences, Tehran, Iran; 9 Department of Epidemiology, School of Public Health, Sahid Beheshti University of Medical Sciences, Tehran, Iran

**Keywords:** Blood pressure percentile, age, body mass index, nomograms, adults

## Abstract

Since no comprehensive study has been conducted on blood pressure (BP) percentiles established upon nationally representative sample population of adults, the present study aimed to construct the blood pressure percentiles by age, sex and body mass index (BMI) of the subjects. Analyses were based on data collected in 2011 from 8,425 adults aged 25 to 69 years old. Data on demographic characteristics, anthropometric measurements, and blood pressure was recorded for each subject. Linear Regression analysis was used to assess the adjusted relationship of age-sex-specific standard deviation scores of BMI, height, and weight with blood pressure. Four separate models for systolic blood pressure (SBP) and diastolic blood pressure (DBP) of men and women were constructed for BP percentiles according to age and BMI. Blood pressure increased with the rise in BMI and weight, but showed a negative correlation with height. SBP and DBP rose steadily with increasing age, but the rise in SBP was greater than DBP. Overweight and obese population, seem to fall into the category of hypertensive. The findings of present study show that BP percentiles are steadily increased by age and BMI. In addition, most obese or overweight adults are hypertensive.

## Introduction

A great deal of the worldwide burden of disease is attributed to high blood pressure, including about 13.5 % of the premature deaths and 6 % of the total global Disability Adjusted Life years (DALYs) (Lawes et al., 2008[14]). Therefore, hypertension in adults and children has drawn attention of many researchers and controlling it has become as a worldwide priority among health policies. Because early detection of hypertension can help control its various complications, blood pressure measurement is now regarded as an important part of routine physical examination (Somu et al., 2003[19]). To interpret measurements precisely, they should be compared to the standard blood pressure (BP) nomograms. However, the distribution of BP values vary based on ethnicities and races, thus standard values derived from a specific population might not be applicable to others; consequently, local reference data could be a better choice to evaluate the BP measurements properly (Goonasekera and Dillon. 2000[6]). In addition, several studies show that overweight and obesity are associated with higher levels of blood pressure and subsequently higher prevalence of hypertension (Robinson et al., 2004[18]; McGavock et al., 2007[15]; Hosseini et al., 2010[10]). So, it is important for each population to develop national BP percentiles for age, sex, and potential anthropometric characteristics which may influence BP levels. In recent years few studies have been conducted to develop blood pressure nomograms for age and anthropometric characteristics, simultaneously, only for children and adolescents (Neuhauser et al., 2011[16]; Yan et al., 2013[22]). But none of them have evaluated adult populations. Janghorbani et al. in 2007[12] reported age-adjusted BP for Body Mass Index (BMI) categories based on a nationally representative population of 89404 subjects. However, they only presented the mean of systolic blood pressure (SBP) and diastolic blood pressure (DBP) and did not model BP percentile for adults' population according to age and BMI, simultaneously. In 2009, Esteghamati et al.[4] reported that the prevalence of hypertension to be 25 % in a population with 5287 subjects (15-64 years), based on the seventh report of the joint national committee on prevention, detection, evaluation, and treatment of high blood pressure (JNC-7) guideline. Moreover, a meta-analysis by Haghdoost et al. in 2008[7] also reported the prevalence of 23 % for adults aged 30-55 years and 50 % for subjects older than 55 years. Therefore, this study aimed to present blood pressure percentiles by age, sex, and BMI of a nationally representative sample population of adults.

## Materials and Methods

### Study population

Data were collected by Iran’s Center for Disease Control and Prevention through the sixth round of national surveillance of Non Communicable Diseases Risk Factors in 2011. A sample of 8425 Iranian adults aged between 25 to 69 years was chosen by a multi-stage cluster random sampling scheme. Fifty counties were randomly selected as the primary sampling units by using the systematic proportional-to-size probability technique. Each of these units included twelve clusters each containing 20-person, chosen via the same method from rural and urban areas. Households were the sample listing units. A maximum of two persons, one younger than 55 years and the other older than 55 years, were selected in each household using a KISH randomization method. 

The study questionnaire, initially proposed by World Health Organization and validated in Iran, was completed for each subject through a face-to-face interview by trained staff from 51 medical schools across the country. An informed consent was obtained from the subjects. The study was approved by local Ethical Committee. 

### Measurements

#### Blood pressure measurements

The blood pressures were measured after at least a 5 minute rest by a standard mercury sphygmomanometer (Model 1002/Presameter, Riester, Germany) with the subjects in a comfortable sitting position, a wakeful state and the right arm positioned at the heart level. 

The selected cuff had a bladder with the width of approximately 40 % of the arm length, long enough to cover 80 % - 100 % of the arm circumference. The cuff was placed 1 inch above the antecubital fossa, and inflated to occlude the wrist pulse. With the stethoscope placed over the brachial artery, the cuff was deflated. The first Korotkoff (K1) sound was considered to be correlated with the systolic BP and the fifth phase of Korotkoff sounds with the diastolic BP. BPs were measured twice with an interval of at least 30 seconds and the mean of the two recordings was used for data analysis. The differences were insignificant. 

#### Height and weight measurements 

The subjects’ height (cm) was measured barefoot in a standing upright position with the heels and back against a vertical scale, using a stadiometer (Seca Model 207 Germany). To measure the weight, a balanced scale (Seca Model 710 Germany) was used with the subject barefoot without heavy outer clothing.

BMI was calculated as body weight (kg) divided by the square of the subject’s height (m). Having passed a training course for anthropometric measurements, a team of field technicians in each administrative district measured all the required data.

### Age-sex-specific standard deviation (SD) scores

To compare the relationships of BMI, weight, and height with blood pressure we used the following formula to change them into age- and sex-specific SD scores (or z-scores): 


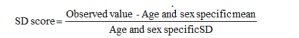


The relationship between each variable and blood pressure was described as the change in BP in response to an increase of the variable by 1 SD. Linear regression analysis was used to estimate the changes in blood pressure. The coefficient of this analysis represented the estimated change in response to 1 SD increase. The changes in BP in relation to BMI were calculated for men and women, separately, as well as for each of the age- and sex-specific groups. To assess the relationship between BP and weight, the height was adjusted and for its relationship with height, the weight was adjusted, as well. 

### Construction of the BP level nomograms according to age, sex, and BMI

Four separate models for SBP and DBP of men and women were constructed for BP percentiles as a function of both age and BMI. First, the Lambda-Mu-Sigma (LMS) method was used to model the BMI percentiles with age for men and women. Then, the reference curves for adults by age and BMI were fitted, simultaneously, using an extension of the LMS method for two covariates. The generalized additive models for location scale and shape (GAMLSS) with the Box-Cox-Cole- Green distribution family (Rigby and Stasinopoulos, 2005[17]; Stanojevic et al., 2008[20]; Cole et al., 2009[3]) fitted with GAMLSS 4.2-0 in the free statistical software R 2.15.2 (http://www.R-project.org). Assumption of normality or a constant variance of BP values with age and/or BMI was not required for neither of them. The skewness parameter L, the median M, and the coefficient of variation S, were modeled as a function of age and/or BMI either as polynomials or non-parametrically by cubic splines. The generalized Akaike information criterion and the percentage of data outside the smoothed percentiles were investigated to examine the goodness of fit. 

In LMS modeling of BMI with age, the skewness parameter L, the median M and the coefficient of variation S, were 2, 3, 3, both for men and women. 

Median of SBP varied with age and BMI. Median of DBP was given by a quadratic function in age and linear in BMI. For SBP in men and women, the model of medians included cubic spline of age, a linear term for BMI, the interaction terms BMI×age, and BMI×age2. Similar models were used for the median of DBP in both genders; however, for the DBP of women, the interaction term of BMI×age2 was excluded as non-significant. Coefficient of variation (S) of SBP and DBP varied as a linear function of age. 

In this study, only the mentioned percentiles were reported, but any (100α) percentile Pα can be calculated by the following formula: 


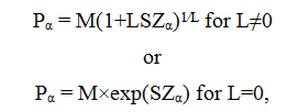


with Z_α_ the α quantile of a standard normal distribution. 

## Results

Blood pressure was measured in a nationally representative sample of 8,425 adults aged 25 to 69 years old in Tehran. Table 1(Tab. 1) shows the baseline demographic characteristics of the study population. Of the 3,381 male subjects, 1289 (38.1 %) subjects were aged 55-69, 970 (28.7 %) aged 25 to 34, 607 (18.0 %) aged 35-44, and 515 (15.2 %) aged 45-54. The studied population included 5044 women, 1705 (33.8 %) of whom were aged 55 to 69, 1409 (27.9 %) subjects were 25 to 34 years old, 983 (19.5 %) 45 to 54, and 947 (18.8 %) aged between 35 to 44 years old. 

The mean of BMI among men was highest in the age group of 55 to 69 with 26.3 kg/ m^2^. Unlike the men, mean of BMI in women did not increase with age and the highest mean of BMI was calculated 29.0 kg/m^2^ for the age group of 45 to 54. It was 28.6 kg/m^2^ for the age group of 55 to 69. 

The mean of SBP and DBP measurements in both genders increased with age. Except for the age group of 25 to 34, the mean of DBP measurements was higher in women compared to men. On the other hand, men had higher SBP means in all age groups except for the age group of 55 to 69. 

950 men were categorized as hypertensive; that is 28.1 % (95 % CI: 26.6 % -29.6 %) of the study population. 1478 (29.3 %) female subjects were categorized as hypertensive (95 % CI: 28.0 %-30.6 %). 

The effects of BMI, weight and height on BP are shown in Table 2(Tab. 2). As can be seen, BP increased with the rise of BMI and weight, but it showed a negative correlation with height. Systolic BP increased 2.77 and 3.19 mmHg for women and men, respectively, with 1 SD increase in BMI when adjusted for age. Similarly, diastolic BP increased 1.69 and 2.28 mmHg with 1 SD increase in BMI. After adjusting for height and age, weight remained to be substantially associated with BP. We found an increase of 2.64 mmHg for systolic BP and 1.77 mmHg for diastolic BP in women, and 3.66 mmHg for systolic BP and 2.56 mmHg for diastolic BP in men, corresponding to 1 SD increase in weight. However, 1 SD increase in height found to decrease systolic and diastolic BP 1.50 and 1.09 mmHg in women and 0.88 and 0.99 mmHg in men, respectively, when it was adjusted for weight and age. All 95 % confidence interval (CI) of the above estimates of effect were narrow, as shown in Table 2(Tab. 2).

The standardized coefficients (|t|) of BP in relation to BMI in women were higher than the coefficients of its relation to weight. As for the men these coefficients were similar for BMI and weight. Since the interpretation of BP norms according to BMI (obesity) seems to be more convenient, it was decided to present blood pressure percentiles by age and BMI for adults.

Tables 3(Tab. 3) and 4(Tab. 4) demonstrate the 50^th^, 90^th^, 95^th^, and 99^th^ percentiles of SBP and DBP for the 5^th^, 10^th^, 25^th^, 50^th^, 75^th^, 90^th^, and 95^th^ percentiles of BMI in specific selected ages of both genders. To create a better perception of data presented in Tables 3(Tab. 3) and 4(Tab. 4), four line graphs were designed to show the trend of changes in 90^th^, 95^th^, and 99^th^ of SBP and DBP percentiles by age for the 75^th^ and 95^th^ BMI percentiles of men and women. The 75^th^ percentile of BMI for the subjects aged 25 to 70 years old ranged from 25.87 to 29.08 kg/m^2^ for men and 26.86 to 31.94 kg/m^2^ for women. 

**Figure 1:**
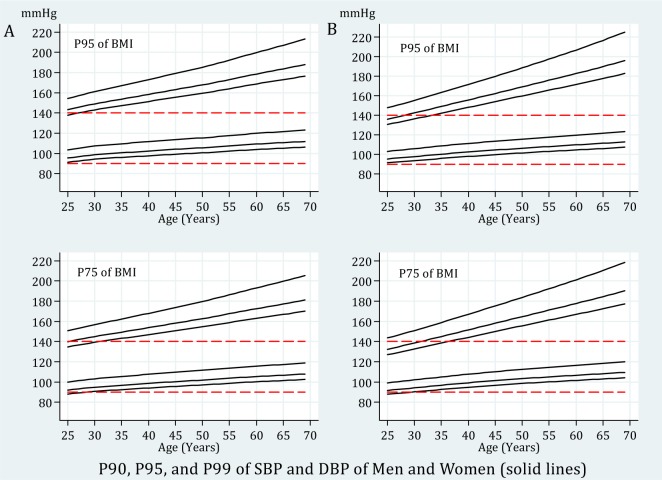
Trend of 90^th^, 95^th^, and 99^th^ BP percentiles by age for 75^th^ and 95^th^ BMI percentiles. A: men; B: women

Therefore, the 75^th^ BMI percentile of men was categorized as overweight and for the women categorized as overweight or obese class I. Moreover, the corresponding values for the 95^th^ percentile of BMI were 30.59 to 33.61 kg/m^2^ for men and 32.83 to 36.98 kg/m^2^ for women, being classified as obese class I or II. According to the JNC-7 report (Chobanian et al., 2003[2]), the cut off values of 140 mmHg for SBP and 90 mmHg for DBP are shown as red lines in graphs for comparison. While both SBP and DBP rose steadily with increasing age, the rise of SBP was greater than DBP (Figure 1(Fig. 1)). Most of the adults with BMIs classified as overweight and obese seem to be categorized as hypertensive by the JNC-7 definition. 

## Discussion

The present study showed that both the SBP and DBP rose steadily with increasing age, for BMI percentiles. Wright et al. in 2011[21], depicted the rise in mean of SBP and DBP with age in adults 18 years and older in the United States during 2001 to 2008. The rise of SBP with age found in this study, is congruous with the results of their study except for the amount of changes observed with increasing age; such increase seems to be much greater in our population (nearly 30 mmHg) compared to the American population (at most 15 mmHg). As for the DBP, their results demonstrated a curvilinear trend with increasing and then decreasing means with age in both men and women. On the contrary, we found the mean of DBP to be steadily rising with increasing the age. If we had categorized our data like their survey into three groups of untreated hypertensive, treated hypertensive and normal, we could have assessed the possibility of treatment differences being responsible for the disagreement observed between these two studies. 

According to the cut off values of 140 mmHg for SBP and 90 mmHg for DBP in diagnosis of hypertension presented in the JNC-7 report in 2003 (Chobanian et al., 2003[2]), 26.4 % of the adult population were estimated to have hypertension (He and MacGregor. 2007[9]). This figure is predicted to increase from 1 billion worldwide, to 1.56 billion by 2025 (Kearney et al., 2005[13]). Considering the same cut off values in the present study, the 140 mmHg SBP is approximately at our 80^th^ SBP percentile and the 90 mmHg DBP also nearly at 80^th^ DBP percentile.

As mentioned above, differences in ethnicity, diet, climate, and many other factors can impact the risk of developing hypertension in a population. So it seems reasonable to produce reference blood pressure percentiles for each population individually. In present study, weight, height, and BMI relationship with BP measures were assessed after adjusting for potential confounders and found that the most convenient variable would be BMI; not only because of its stronger association with blood pressure, but also because it is a better representative for some of the factors believed to have an impact on blood pressure such as diet. In evidence based guideline of 2014 for management of high blood pressure in adults, the panel members appointed to the JNC-8 made nine recommendations as when to start medical treatment in hypertensive patients (James et al., 2014[11]). They did not redefine the high blood pressure of 140/90 presented in JNC-7, they recommended to start pharmacological treatment to lower blood pressure at SBP ≥ 150 mmHg or DBP ≥ 90 mmHg in the general population aged ≥ 60 years. So, changing the cut off values is probable for each population, based on the characteristics and specifications of their people. 

The prevalence of hypertension (SBP > 140 or DBP > 90) in Iranian population is high with a total prevalence of 28.8 % that specially increases in old ages. This is similar to the total prevalence of hypertension in the American population older than 18 years, reported as 30.4 % (CDC, 2012[1]). Since the mortality rates of ischemic heart diseases (26 %) and CVAs (10 %) in Iranian population are also similar to the data from developed countries (Forouzanfar et al., 2014[5]), the cut off values of 140 for SBP and 90 for DBP seem to be suitable for our population as well. This high prevalence of hypertension should be a wakeup call for the healthcare system authorities in Iran to take measures in order to prevent the increasing rate of hypertension and its consequent cardiovascular diseases. First level prevention and the most important step is to control for these risk factors. Primary care can also create a foundation for new treatments and medications to control hypertension. These measures can decrease the mortality rate of cardiovascular diseases greatly (He et al., 2005[8]). Such measures are the reason for the decrease in burden of cardiovascular diseases in developed countries during the past few years.

In this study we only presented the nomograms of blood pressure and developed BP percentiles by age, gender, and BMI. But, for these findings to be applicable in medical practice and to be utilized in developing reference values and cut offs for hypertension, they need to be compared with the results of other studies assessing the relation between the risk of cardiovascular diseases with blood pressure. Lack of such studies in Iran limits our ability to interpret the results of the present research. 

## Conclusion

The present study, for the first time, constructs BP standard curves based on age and BMI centiles in Iranian adults. Our results show that BP percentiles steadily increased by age and BMI. In addition, most obese or overweight adults are hypertensive. These findings strongly support the need for screening of hypertension in overweight and obese population.

## Acknowledgement

We would like to thank the non-communicable diseases risk factor surveillance Office, Center for noncommunicable diseases Control & Management, Center for Disease Control (CDC) and Iranian Ministry of Health and Medical Education for providing this invaluable data. This study was supported by I.R. Iran’s National Institute of Health Research, Tehran University of Medical Sciences. Contract No: 241-M-9309.

## Conflict of interest

None.

## Figures and Tables

**Table 1:**
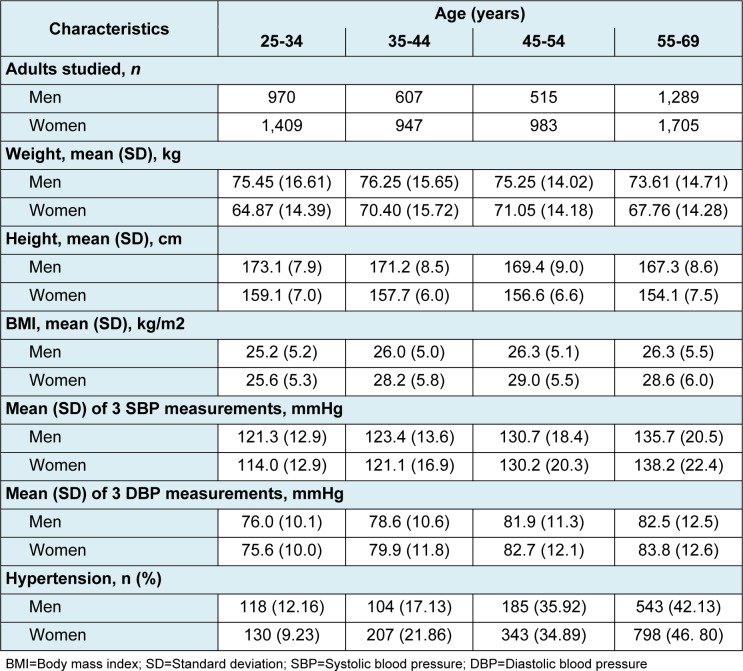
Baseline characteristics of the adults study population (3381 men and 5044 women aged 25 - 69 years)

**Table 2:**
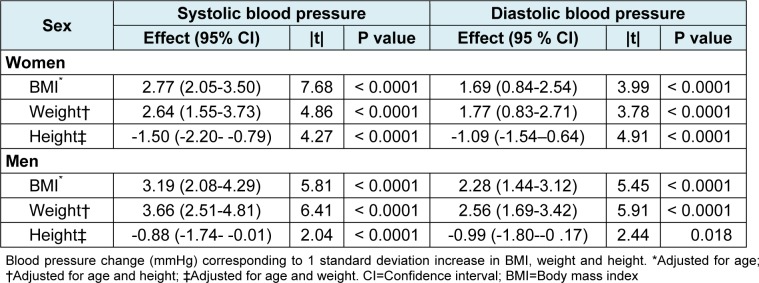
Effects of body size on systolic and diastolic blood pressure measurements

**Table 3:**
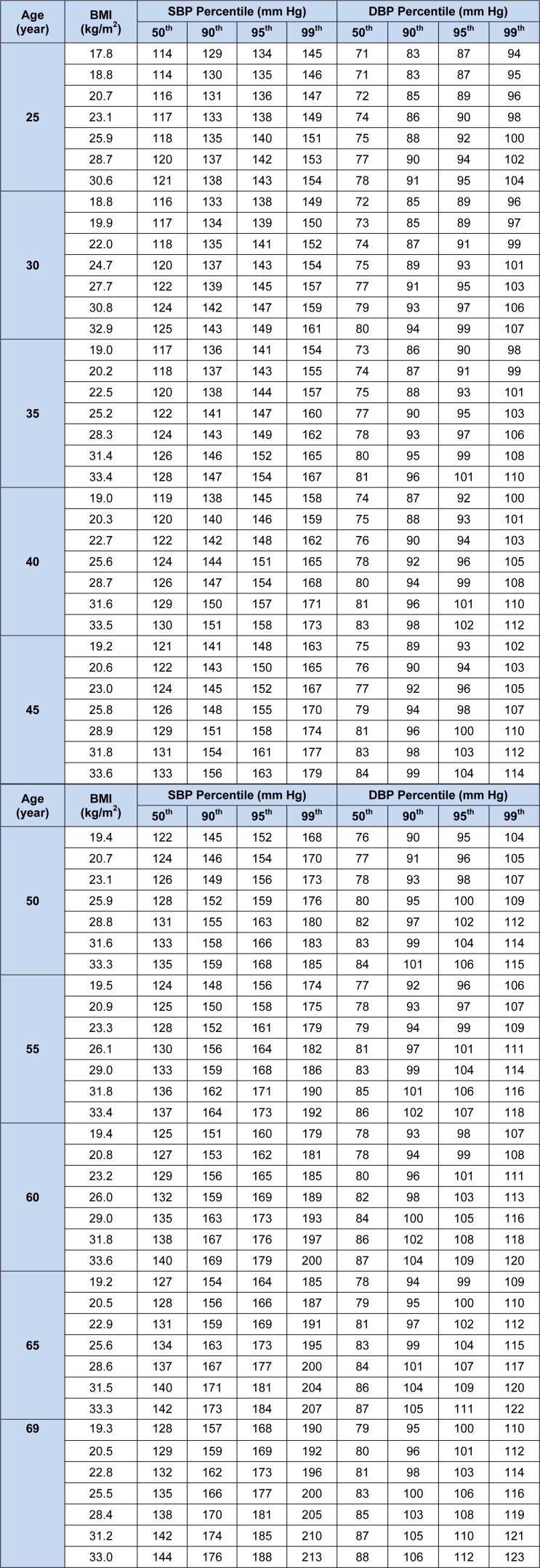
BP Levels for males according to age and BMI

**Table 4:**
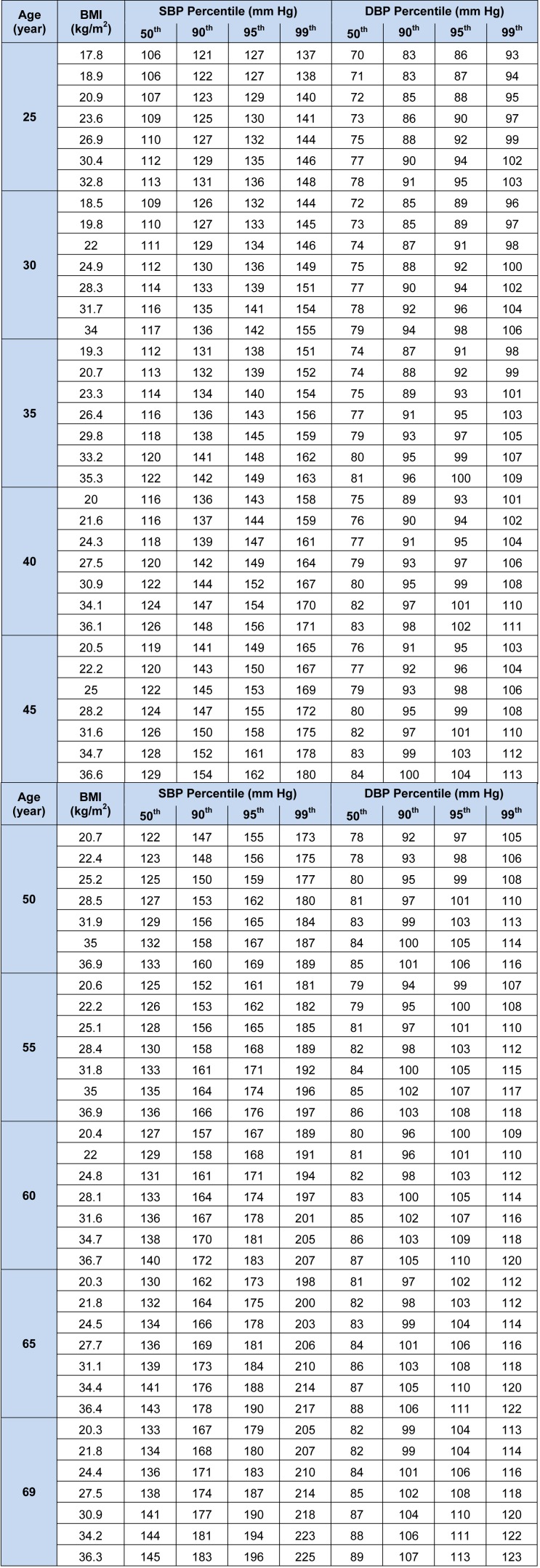
BP Levels for females according to age and BMI
